# Sinonasal neuroendocrine carcinoma: impact of differentiation status on response and outcome

**DOI:** 10.1186/1758-3284-3-32

**Published:** 2011-07-27

**Authors:** Anna Likhacheva, David I Rosenthal, Ehab Hanna, Michael Kupferman, Franco DeMonte, Adel K El-Naggar

**Affiliations:** 1 Department of Radiation Oncology, The University of Texas MD Anderson Cancer Center, 1515 Holcombe Blvd, Houston, TX 77030, USA; 2 Department of Radiation Oncology Treatment, The University of Texas MD Anderson Cancer Center, 1515 Holcombe Blvd, Houston, TX 77030, USA; 3 Department of Head and Neck Surgery, The University of Texas MD Anderson Cancer Center, 1515 Holcombe Blvd, Houston, TX 77030, USA; 4 Department of Head and Neck Surgery, The University of Texas MD Anderson Cancer Center, 1515 Holcombe Blvd, Houston, TX 77030, USA; 5 Department of Neurosurgery, The University of Texas MD Anderson Cancer Center, 1515 Holcombe Blvd, Houston, TX 77030, USA; 6 Department of Pathology, The University of Texas MD Anderson Cancer Center, 1515 Holcombe Blvd, Houston, TX 77030, USA

**Keywords:** Neuroendocrine carcinoma, carcinoid tumor, poorly differentiated carcinoma, sinonasal tumor

## Abstract

**Background:**

The impact of tumor differentiation on the behavior and response of sinonasal neuroendocrine carcinoma is unknown.

**Methods:**

We performed a retrospective review of the patients treated for neuroendocrine carcinoma (NEC) of the nasal cavity or paranasal sinuses from 1992 to 2008 at MDACC.

**Results:**

The results of our study suggest that pathologic differentiation may not be a critical factor in the clinical management of patients with NEC of the sinonasal tract. This is in contrast to laryngeal and lung NEC for which pathological differentiation has traditionally guided clinical management.

**Conclusion:**

Mutlimodality approach should be the cornerstone of treating sinonasal NEC regardless of their differentiation. Specifically, RT may provide durable local control for patients with moderately differentiated NEC if resection is not feasible or desirable, while surgical resection can benefit patients with chemo-resistant or radio-resistant disease.

## Introduction

Neuroendocrine neoplasms are classified into well-differentiated (typical), moderately differentiated (atypical carcinoids), and poorly differentiated (small and non-small cell types). Well- and, to a lesser extent, moderately differentiated neuroendocrine carcinomas carry better prognosis with low metastatic rates and better survival [1-4], while poorly differentiated neuroendocrine carcinomas are characterized by rapid and fatal outcome prognosis. Treatment recommendations for this entity vary considerably due largely to a lack of consensus and variable pathological classification.

The majority of neuroendocrine carcinomas in the head and neck region arise in the larynx and constitute the second most common malignancy after squamous carcinomas [5,6]. The treatment of laryngeal neuroendocrine carcinomas is generally based on tumor differentiation status where well and moderately differentiated tumors are treated surgically and the poorly differentiated are managed by radiation and/or chemotherapy [6-9]. Primary neuroendocrine carcinomas of the sinonasal tract are rare and represent a histological spectrum of differentiation [10-12]. Because of their infrequency and overlapping pathologic features with other entities, studies investigating the effect of differentiation status on clinical behavior and management of patients with these tumors remained unaddressed [10].

We present a series of 20 patients with neuroendocrine carcinoma of paranasal sinuses at MD Anderson Cancer Center to evaluate whether differentiation may affect treatment and outcomes of these patients.

## Patients and Methods

We performed a search of the head and neck pathology, surgery and radiation oncology databases at the University of Texas M. D. Anderson Cancer Center for patients diagnosed and treated for neuroendocrine carcinoma of the nasal cavity or paranasal sinuses from 1992 to 2008. A waiver of consent was obtained from the Institutional Review Board and patient confidentiality preserved. Information was obtained by retrospective chart review from the in-house electronic medical records system. The clinicopathological, treatment and follow-up information were reviewed. Some of the patients received their entire course of treatment at our institution, while others were initially treated at other facilities and underwent salvage therapy at MD Anderson. A total of 41 patients treated for neuroendocrine carcinoma of the paranasal sinuses were identified. 21 patients were excluded due either to loss to follow up or lack of the pathological materials. Twenty patients with available diagnostic materials and full records for follow-up comprised the cohort of this study. All hematoxylin and eosin and immunostained markers for keratin and endocrine markers were reviewed independent of the original diagnosis. Complete agreement between the initial diagnosis and the re-evaluation that the selected case represent NECs. Sites of primary malignancy were specifically limited to nasopharynx, nasal cavity, skull base, maxillary, ethmoid, and sphenoid sinuses. The patients' records were reviewed for the following parameters: age, sex, race, symptoms, radiological features, presenting symptoms, site of tumor and local extension, surgical approaches and extent of resection, adjuvant therapies, treatment complications, and patient outcomes, including dates of death, last follow-up, and local, regional, and distal failures. Patients were staged according to the American Joint Committee on Cancer (AJCC) TNM 2002 staging classification of the nasal cavity and the paranasal sinuses. Treatment modalities for each individual patient at MDACC were determined by a multidisciplinary physician team based on clinicopathological features and prior treatment, if present. The extent of surgery was assessed for presence of complete gross resection vs. subtotal resection. Locoregional failure was defined as relapse at primary sites or contiguous lymphatic drainage basins. Disease free survival was calculated from the last date of treatment to either locoregional or distant failure or date of last follow-up if there was no evidence of failure. Overall survival was calculated from the last date of treatment to either date of death or date of last follow-up if patient was still alive.

## Results

### Patient demographics

Table 1 presents the clinical and demographic information on patients with sinonasal neuroendocrine carcinoma. There were 11 men and 9 women, with a mean age of 49.2 years. For those patients in whom this information was available, mean time from clinical signs to diagnosis was 10 months, and initial symptoms included: nasal congestion (n = 10, 50%), epistaxis (n = 6, 30%), headache (n = 3, 15%), persistent sinusitis (n = 3, 15%), and neck mass (n = 2, 10%) (Table 1). In the group of patients with poorly differentiated NE carcinoma, one patient had reported receiving radiation to the head and neck in childhood for tonsillitis, and another patient had a retinoblastoma gene mutation with a previous diagnosis of bilateral retinoblastomas and breast cancer.

**Table 1:** Patient demographics and outcome

Pt	Sex/age	Differentiation	TNM (stage)	Surgical Resection	Chemotherapy	Radiotherapy	Failure (mo)	Definitive Treatment of recurrence	Follow-up (mo)
1	M/22	mod	T4aN0M0 (IVA)	ND, MM, mandibulectomy	none	AdRT	none	none	NED (166)
2	F/67	mod	T4aN2bM0 (IVA)	CFR, R ND (I-III)	NAC	AdRT	dural metastases (21)	none	DOD (56.2)
3	F/43	mod	T1N0M0 (I)	SPH, ETH	none	AdRT	none	none	NED (31.3)
4	M/46	mod	T4bN1M0 (IVb)	none	NAC	CRT	none	none	NED (16.6)
5	M/54	mod	T4N0M0 (IV)	STR ES	Concurrent	AdCRT	Bilateral cervical LN (46)	ND, AdRT	NED (107)
6	M/47	mod	T4bN0M0 (IVB)	STR CFR	none	AdRT	Ipsilateral level I (125)	ND, AdCRT	NED (172)
7	M/54	mod	TxN0M0	S	AdC	AdRT	local (80)	NAC/RT/AdC, CFR	DOD (119)
8	M/78	poorly	T4bN0M0 (IVB)	S	none	none	none	none	NED/Dead (77)
9	F/24	poorly	T4bN0M0 (IVB)	none	NAC; AdC	RT	none	none	NED (136)
10	F/47	poorly	T4bN1M0 (IVB)	none	NAC	RT	leptomeningeal dz (4.1)	none	DOD (13)
11	F/38	poorly	T4bN0M0 (IVB)	CFR	none	AdRT	none	none	NED (47.5)
12	M/53	poorly	TxN0M0	STR ETH	AdC; concurrent	AdCRT	none	none	NED (17.8)
13	M/51	poorly	T3N0M0 (III)	none	NAC; AdC	RT	persistent (0)	none	DOD (17)
14	F/57	poorly	T4aN0M0 (IVA)	CFR, SPH	NAC	none	none	none	DOC (16)
15	M/70	poorly	T4bN0M0 (IVB)	STR ETH	AdC	none	persistent (0)	CFR/RT	DOD (16)
16	F/33	poorly	T2N0M0 (II)	CFR	none	AdRT	Ipsilateral levels I-V (5)	ND, AdRT	NED (149)
17	M/38	poorly	T2N0M0 (II)	none	NAC	RT	local (11)	ETH/MM/SPH, AdCRT	DOD (37.5)
18	F/60	poorly	T4bN0M0 (IVB)	STR CFR	AdC	AdRT	persistent (0)	AdC	NED/Dead (112)
19	F/38	poorly	T4aN0M0 (IVA)	CFR	none	AdRT	none	none	NED (168)
20	M/64	poorly	T4bN0M0 (IVB)	STR CFR	Concurrent	AdCRT	none	none	NED (63)

AWD = alive with disease; NED = no evidence of disease; DOD = dead of disease; DOC = dead of treatment complications. S = resection of unknown type, RT = radiation therapy, AdRT = adjuvant radiation therapy, NAC = neoadjuvant chemotherapy, AdC = adjuvant chemotherapy, AdCRT = adjuvant chemoradiation. CFR = Craniofacial Resection, ETH = Ethmoidectomy, SPH = Sphenoidectomy, MM = Medial Maxillectomy, STR = Subtotal Resection.

### Pathologic evaluation

**Figure 1:**
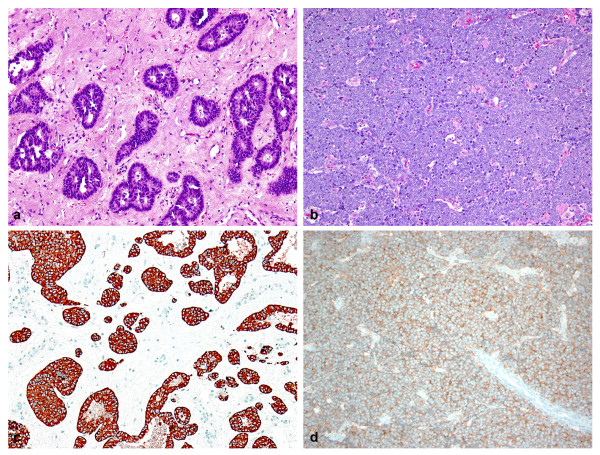
**Photomicrograph of a moderately differentiated (A) and poorly differentiated (B) neuroendocrine carcinomas of the sinonasal tract**. Figure 1C shows a chromogranin positivity in the cytoplasm of tumor cells of a MDNEC and Figure 1D displays synaptophysin positive PDNEC.

The independent histopathologic evaluation focusing on differentiation classified seven as moderately differentiated (atypical carcinoid) and 13 as poorly differentiated (small and non-small cell). (Table 1, Figure 1). Atypical carcinoids were characterized by formation of glandular, organized cell nest or cords of tumor cells. Tumors also manifested cellular pleomorphism, increased mitotic figures and occasional small areas of necrosis. In contrast, poorly differentiated tumors were lacking any differentiation. Both subtypes were positive for keratin and at least one of neuroendocrine markers.

### Stage

Of the patients with MDNEC, one (14%) had stage I (T1N0M0), 5 patients (71%) presented with stage IV, and one (14%) had an Tx stage due to an outside resection (Tables 1 and 2). Two (29%) presented with nodal disease (N2b and N1). Patients with PDNEC were staged as follows: two (15%) had stage II (T2N0M0), one (8%) had stage III (T3N0M0), nine (69%) presented with stage IV (six with T4bN0M0, two with T4aN0M0, one with T4bN1M0), and one (8%) had an unknown stage due to an outside resection. Only one patient (8%) presented with nodal disease (N1).

**Table 2:** Characteristics, presentations, and outcome of patients with sinonasal moderately differentiated (MDNEC) and poorly differentiated (PDNEC) neuroendocrine carcinoma

Characteristic	MDNEC	PDNEC
*Demographics*		
Age at presentation	47.6 (22-67)	50 (24-78)
M:F ratio	5:2	6:7
*Major Presenting Symptoms*		
Congestion/Sinusitis	3 (43%)	7 (54%)
Epistaxis	3 (43%)	3 (23%)
Hearing loss	1 (14%)	0
Neck mass	0	2 (15%)
*Stage*		
I	1 (14%)	0
II	0	2 (15%)
III	0	1 (8%)
IV	5 (71%)	9 (69%)
DFS		
Ch + RT	11 (mon)	7.6 (mon)
RT + Surgery	63 (mon)	16.0 (mon)

• Ch: Chemotherapy

• MDNEC: Moderately differentiated

• PDNEC: Poorly differentiated

• RT: Radiotherapy

### Treatment modalities

Multimodality approach, using surgical resection, systemic chemotherapy and radiation, was used in the treatment of most patients (Table 2) Surgical treatment included craniofacial resection (n = 8, 40%), medial maxillectomy (n = 2, 10%), ethmoidectomy (n = 3, 15%), and sphenoidectomy (n = 2, 10%). Neck dissection was performed in 2 patients with nodal metastasis. For two of the patients who underwent surgery the specific type of resection was not known due to lack of available records. Of the 15 patients undergoing surgical resection, subtotal resection was documented in 6 patients (40%). The resection of one poorly differentiated neuroendocrine carcinoma was complicated by a CSF leak, pneumocephalus, and pontine hemorrhage, which led to the eventual demise of the patient after a protracted illness. No other major complication was noted after surgical procedures.

Chemotherapy was administered in neoadjuvant, concurrent and adjuvant settings, as well as part of salvage treatment. Chemotherapy treatment included cisplatinum and etoposide (n = 9), carboplatin and etoposide (n = 4), cis-platinum and 5-fluorouracil (n = 2), and Taxotere (n = 1).

Radiatiotherapy (RT) was administered as part of definitive, adjuvant or salvage treatment. The technique and doses varied in part because of the long span of treatment years. Unfortunately, treatment details were not available in some cases because the treatment took place at an outside facility or before electronic medical record system was instituted. The available treatment details indicated that radiation was delivered by either 3D conformal radiation therapy or intensity modulated radiation therapy. All patients with the exception of patient 6 who did not finish his prescribed course, were treated to at least 60 Gy.

### Outcomes

Follow-up interval for surviving patients ranged from 13 to 172 months (median 60 months). Recurrences were predominantly locoregional, with only one patient in each group having distant metastases. 4 patients (57%) in MDNEC group and 6 patients (46%) in PDNEC group suffered from recurrences. In the MDNEC group, patient 7 had local recurrence at 80 months. Patients 5 and 6 had cervical lymph node (LN) recurrence at 46 and 125 months, respectively. Patient 2 had dural metastases identified at 21 months. In the PDNEC group, patients 13, 15 and 18 had persistent disease with no radiographic evidence of remission. Patient #17 had local recurrence at 11 months and patient #16 suffered from cervical LN recurrence at 5 months. Median overall disease free survival (DFS) for both groups was 23.6 months while overall survival (OS) was 58.6 months.

#### MDNEC

The median DFS and OS were 46 months and 107 months respectively. All patients received radiation therapy as part of their treatment. Those patients, for whom resection was part of management, had a median DFS of 63 months and a mean OS of 113 months (Table 2). Those patients with subtotal resections had a median DFS of 85 months and a mean OS of 139 months. 5 patients (71%) were alive at last follow-up. The patient with the poorest DFS and OS (11 and 17 months respectively) was the patient that did not have a surgical resection, and instead received definitive CRT (Table 2).

#### PDNEC

The median DFS and OS were 11 months and 47.5 months respectively. Ten out of thirteen patients received RT as part of their treatment and their median DFS was 13.3 months and OS was 55 months. The four patients who did not undergo surgery but instead received definitive chemoradiation had a median DFS of 7.6 months and OS of 27.3 months (Table 2). The nine patients who received surgery as part of their treatment had a median DFS of 16 months and a mean OS of 63 months. 8 patients (62%) were alive at last follow up.

## Discussion

Sinonasal NEC is a rare malignancy, the clinical behavior of which is not well known. The paucity of published literature on this subject had been compounded by the fact that previous studies of sinonasal NEC have included a subset of broad spectrum of neuroectodermal tumors, including olfactory neuroblastoma, small cell neuroendocrine carcinoma and sinonasal undifferentiated carcinoma. Management decisions for these rare sinonasal NEC's often have to be based on analogous treatment principles for NEC of other anatomical sites, such as lung and larynx, where histopathological differentiation is generally considered a key element to guide therapeutic decisions. To the authors' knowledge, this is the first paper to compare clinical history, recurrence patterns, and treatment outcomes between moderately and poorly differentiated sinonasal NEC. Focused pathologic review was conducted to determine the neuroendocrine differentiation was accurate status of these tumors.

Multimodality approach was utilized in treatment of most patients at our institution regardless of the level of differentiation. Only one patient in the MDNEC group did not undergo surgery as part of his initial treatment, and his DFS was much lower (11 months) compared to the rest in the group who underwent a resection (63 months) (Table 2). In the PDNEC group, nine patients (69%) underwent surgical resection as part of initial treatment and their DFS was also greater compared to those in the group who did not, 16 vs. 7.6 months (Table 2). Interestingly, the four patients in the PDNEC group who had persistent disease or local failure had non-surgical based treatment or an incomplete surgical resection. Since surgical intervention in the head and neck is not without substantial risks and morbidity, the extent of surgical intervention should be considered in the evaluation. The role of minimally invasive endoscopic resection was not directly assessed in this study, but utilizing this approach can limit surgical morbidity without compromising oncologic outcomes.

All patients with MDNEC received RT as part of their initial treatment, and all but one patient underwent surgery. Patients with complete local control had all received RT with only one patient who had a local recurrence at 80 months after undergoing surgery with adjuvant RT and chemotherapy. Ten patients (77%) in PDNEC group had RT as part of their initial treatment. Of the three patients who did not have RT, only one had persistent disease and was treated with a re-resection at MDACC and adjuvant chemo-re-irradiation and died of disease. DFS was greater for all patients who underwent resection as part of their initial treatment (Table 2). Our results suggest that pathological differentiation may not be a critical factor in the clinical management of patients with these tumors. In our small series, 1 year, 5 year and 10 year local control rates for neuroendocrine carcinomas were comparable. Although MDNEC manifested a less aggressive course than its poorly differentiated counterpart, our data show that both entities may benefit from a multi-modality approach. Two patients with PD and one with MD NEC developed neck recurrences, and none of these had neck RT, and all three of these patients were salvaged. In contrast, no patient who had neck irradiation developed a local recurrence. This suggests that neck in addition to primary site irradiation should be considered for patients with both MD and PD NEC's. One patient succumbed to leptomenengial disease and another to dural metastases. RT technique may be important to reduce such risk with adequate coverage of adjacent dural pathways of potential spread.

In our study, the majority of recurrences in both groups were locoregional, suggesting that local control may be independent of differentiation though the numbers are small to make a categorical conclusion. A previous study of undifferentiated tumors in the sinonasal region showed that twenty patients with NEC eight suffered from locoregional recurrence while only one patient died of metastatic disease [13]. In a published M.D. Anderson experience with sinonasal carcinomas by Rosenthal et al, locoregional failure for SNEC was 40% while the rate of distant metastases was 14% [1]. It was also concluded that RT and surgery appear to play an important role in treatment of both groups. Although our analysis suggests that differentiation may have a prognostic significance in terms of DFS and OS, the role in the therapeutic response is minimal.

Our findings are in contrast to studies for NEC of the larynx where MDNEC were refractory to radiation and chemotherapy, with surgical resection being the primary method of treatment [5,8]. PDNEC, however, has both high local recurrence and distant metastasis rates, and is radiation-responsive, so traditionally the preferred treatment has been systemic chemotherapy and radiotherapy (RT) [9,13]. Recent studies have demonstrated a benefit of multimodality approach for neuroendocrine carcinomas of the head and neck. A retrospective review of 11 patients with MDNEC of the larynx treated at M.D. Anderson Cancer Center, Gillenwater et al. reported that a subset of these patients' disease responded to radiation and chemotherapy [14]. While PDNEC of the head and neck has traditionally been treated with concurrent chemoradiation because of the propensity for early spread, there are multiple reports in the literature whereby these malignancies were treated successfully with surgery alone [15-18]. A distinction must be made between small cell and non-small cell types of PDNEC. Two studies of combined modality treatment with neoadjuvant chemotherapy and high-dose precision RT for esthesioneuroblastomas and poorly differentiated NEC found that craniofacial resection is effective especially in recurrent or persistent disease [19,20]. Thus, RT may provide durable local control for patients with moderately differentiated NEC if resection is not feasible, while post-RT surgical resection can benefit patients with chemo-resistant or radio-resistant disease. One potential approach that is under study at our institution is the role of induction chemotherapy in stratifying patients for either surgical resection of combined chemoradiotherapy regimens based on response. Further study is necessary to delineate the ideal approach for this disease.

## Competing interests

The authors declare that they have no competing interests.

## Authors' contributions

All authors were involved in the design, interpretation and review of the study. All authors read and approved the final manuscript.
